# Exercise Stress Echocardiography with Tissue Doppler Imaging (TDI) Detects Early Systolic Dysfunction in Beta-Thalassemia Major Patients without Cardiac Iron Overload

**DOI:** 10.4084/MJHID.2012.037

**Published:** 2012-06-14

**Authors:** Umberto Barbero, Paola Destefanis, Roberto Pozzi, Filomena Longo, Antonio Piga

**Affiliations:** 1 Cardiology Division, San Luigi Gonzaga Hospital, Orbassano, Italy; 2 Thalassemia Center, University of Turin, Orbassano, Italy

## Abstract

**Objectives:**

To evaluate left and right myocardial performance at rest and after maximal exercise by conventional and Tissue Doppler Imaging (TDI) echocardiography.

**Background:**

Iron Overload Cardiomyopathy (IOC) is the main cause of death in thalassemia major (TM) patients but conventional Echocardiography fails to predict early cardiac dysfunction. As TDI is able to demonstrate regional myocardial dysfunction and stress test may reveal abnormalities which are not evident at rest, we wondered if echocardiographic parameters may reveal abnormalities when applied first at rest and then after a physical effort.

**Methods:**

We enrolled 46 consecutive beta-TM patients and 39 control subjects without evidence of cardiac disease; two echocardiograms, at baseline and at the end of maximal exercise on supine bicycle ergometer, were done. All TM patients had a liver iron assessment by SQUID (Superconducting Quantum Interference Device) and a cardiac iron one by MRI (T2*) evaluation.

**Results:**

38 TM patients had no evidence of cardiac iron overload. Whereas TM patients did not shown diastolic dysfunction and all of them presented a good global response to exercise, TDI detected a reduced increase of the S’ waves of left ventricle basal segment during exercise. This finding seems to have some weak but interesting relations with iron overload markers. Pulmonary artery systolic pressure (PAPs) values were greater than in control subjects both at rest and after exercise

**Conclusions:**

in our study, exercise stress TDI-echocardiography was able to demonstrate subtle systolic abnormalities that were missed by Conventional Echocardiography. Further studies are required to determine the meaning and the clinical impact of these results.

## Introduction

Some previous studies reported interesting results about the utility of Tissue Doppler Imaging for the echocardiographic evaluation of patients with beta-talassemia major.^1^ Because all these studies considered echocardiographic evaluation at rest, we wondered if TDI evaluation during exercise stress echocardiography could be more sensitive to early abnormalities. Since a few years, cardiac iron overload evaluation by Magnetic Resonance with T2* technique is available to identify patients at high risk of heart failure and arrhythmia from myocardial siderosis; nevertheless, it is not a fast and bedside technique as echocardiography.

TM patients are prone to iron overload due to chronic blood transfusion and increased intestinal absorption.^2^ Within iron-related complications, iron overload cardiomyopathy (IOC) represents the leading cause of death in these patients.^3^ IOC is characterized by a restrictive pattern with early diastolic dysfunction which progresses to an end-stage dilated cardiomyopathy and heart failure. It is now clear that chelation therapy can prevent, delay or even reverse the signs of cardiac dysfunction, but once overt heart failure is present, only 50% of patients survive.^1^ Early diagnosis of iron toxicity may be crucial to modify iron chelation therapy before heart failure appears, even if symptoms, as well as abnormalities at standard echocardiography, appear late.^4^ The demonstration of a change in contractility of longitudinal myocardial fibers by recent TDI technique could be useful for early detection of regional myocardial dysfunction before the occurrence of abnormal indices of global ventricular function.^5^,^6^

With our study we evaluated the effectiveness of TDI in TM patients without clinical or instrumental signs of heart failure in early detection of regional myocardial dysfunction at rest and after maximal exercise.

## Materials and Methods

### Study Population

From January to October 2011 we examined 46 consecutive patients with beta-thalassemia major (TM) on regular blood transfusion and iron chelation. Inclusion criteria were a recent Biosusceptometry SQUID (Superconducting Quantum Interference Device) to quantify liver iron concentration (LIC) and a cardiac iron assessment by MRI T2* technique in the previous year. Exclusion criteria were: clinical signs of heart failure, pulmonary hypertension, arrhythmias, left ventricular ejection fraction (LVEF) <55%, ongoing pregnancy, age <18 years, reduced exercise capacity or dyspnea at six minutes walking test (6MWT).

We also examined 39 healthy age-, and sex-matched volunteers. The study protocol was approved by local ethics committee/boards, and all patients and control subjects were enrolled after informed consent.

### Echocardiography and Doppler Measurements

Transthoracic echocardiography was performed 4–9 days after blood transfusion to ensure hemodynamic stability, using a Aloka Prosound Alfa 10 ultrasound system (2.5–3.5 MHz), by a single expert investigator that was blinded to clinical information about patients until the end of the study. Cardiac cycles were stored in a digital format. Chamber dimension were determined by standard procedures^7^ and valve flow Doppler and TDI were recorded in the apical 4-chamber view according to guidelines.^8^ Mitral and tricuspid in-flow measurements included peak early (E) and late (A) flow velocities, E/A ratio, deceleration time of mitral E (DT) and right isovolumic relaxation time (IVRT). Systolic (S’), early (E’) and late (A’) diastolic peak velocities of TDI were assessed on three beats at septal and lateral mitral origin and at lateral tricuspid origin, and expressed as a mean. Mean values of the E/E’ and E’/A’ were determined.

Pulmonary arterial systolic pressure (PAPs) was derived from the tricuspid regurgitation velocity and an estimation of the right atrial pressure based on the dimension of inferior vena cava.^9^,^10^

For all measurements, intervals from 3 consecutive cardiac cycles were measured and averaged.^8^

### Stress Echocardiography

During the same session all patients underwent a complete echocardiographic examination (standard and TDI) after maximal exercise performed on a supine ergometer ER-900, Bitz-Germany, increasing workload by 25 Watts every 2 minutes, until they experienced fatigue or symptoms or until 85% of the age-predicted maximum heart rate was achieved.^11^ The ergometer was very close to the ultrasound system allowing execution of echocardiogram at peak of exercise.

### Statistical Analysis

Data are expressed as mean ± standard deviation for continuous variables or count (%) for categoric variables. Variables between the 2 groups were compared using the unpaired Student *t* test or Fisher’s exact test. The degree of association between clinical and laboratory variables were determined using the Pearson’s correlation coefficient. Differences were considered significant at a probability value of p<0.05.

## Results

The characteristics of patients and control subjects are summarized in Table 1. Although patients and control subjects were matched for age and gender, body surface area was significantly smaller in TM patients than in control subjects. Eight TM patients (17.4%) had cardiac iron overload at MRI while 38 had not.

*Echo at rest (*table 3 and 4*):* in patients with TM the morphological data were within normal ranges, with a mild atrial dilation compared to control group (left atria 19.8±3.2 cm^2^ vs 16.8±1.4; p<0.0001). Left atria’s area had a significantly negative relation with mean haemoglobin levels of the previous year (r=0.328; p=0.03).

As regards *systolic function*, in TM patients EF was normal, left S’-waves by TDI were comparable to controls and the average value of Tricuspid Anular Plane Systolic Excursion (TAPSE) was normal (23.9±3 mm).

*Diastolic parameters* were in ranges of normality in both groups, even if right isovolumic relaxation time (IVRT) was higher in TM patients (59.6±19.6 ms) then in the control group (19.7±3.5; p<0.0001).

Also *PAPs* value was within normal limit even if higher in TM patients than in control subjects (24.38±3 mmHg vs 20.8±4.8 mmHg), due to different tricuspid regurgitation jet velocity (TRV) between the two groups (2.18±0.16 m/s vs 1.97±0.3 m/s; p<0.0001). There were no remarkably echocardiographic differences at rest between patients with and without cardiac iron overload detected by MRI (table 6).

*After maximal exercise (*table 5*):* patients showed a good fit, with increase in EF and no signs of ventricular filling pressures’ elevation.

**Figure 1:**
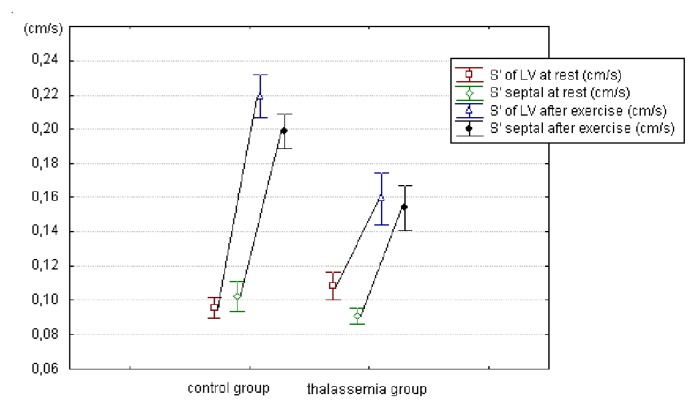
comparison of mean values ± standard deviation of the S’ velocity of the Left Ventricle, at rest and after exercise, in patients with thalassemia (n=46) and in control group (n=39). The difference of the velocity measured after the exercise in thalassemic patients versus control group was highly significant (p<0,0001 for lateral wall’s segment and septum).

S’ waves of the left ventricle increased with stress as expected, but at a significantly lower degree in TM patients than in controls (S’ lateral mitral ring: 0.16±0.05 vs 0.22±0.04 m/sec; p<0.0001 – S’ septal: 0.15±0.04 vs 0.19±0.03; p<0.0001) as showed in figure 1.

Right S’ wave increased more in TM cases than in controls (p<0.0001); also TAPSE, used to evaluate the systolic function of the right ventricle, followed this way, although without statistic significance (p=0.14). TAPSE values significantly differed between patients with and without cardiac iron overload (26.88±3.98 vs 31.26±4.12 mm; p=0.008).

PAPs value increased in both groups, due to TRV rising after exercise (2.63±0.18 m/s in TM patients vs 2.27±0.28 m/s in control group) and remained higher in TM patients (p<0.0001).

## Discussion

Heart disease is till today the major cause of mortality in thalassemic patients.^3^ Iron deposition in myocardial cells induces the production of oxygen free radicals with free fatty acid oxidation, ATP decreased production and Ca^2+^ metabolism abnormalities.^12^ Over that, others factors have been suggested to act in these patients: chronic anemia, resulting in the hyperdynamic circulation; tissue hypoxia; altered coronary regulation and myocarditis;^13^ chronic hemolysis with arginase release and consumption of nitric oxide leading to elevated pulmonary arteriolar resistance and right ventricle dilatation and dysfunction.^14^

Cardiomyopathy from iron toxicity has a silent course, with late onset of symptoms of congestive heart failure,^15^ therefore an instrumental early recognition of cardiac dysfunction may be useful in modifying therapy before cardiac damage occurs.^16^ Cardiac T2* magnetic resonance was demonstrated to have a significant prognostic value in predicting such complications^17^ but, althought giving a strong information about the risks associated with the level of iron load, it does not give an actual knowledge about myocardial damage.

In clinical practice echocardiography provides a fast bedside and relatively cheap technique to study heart function; however, conventional echocardiographic parameters remain normal until late, mainly due to the anemia-related hyperdynamic state. Pulsed Doppler tissue imaging is a relatively new technique which allows the assessment of ventricular regional function, myocardial velocity, time intervals of the cardiac cycle and, most importantly, is preload independent.

There is evidence that long axis function measured by TDI is a more sensitive index of myocardial contractility than conventional echocardiographic parameters. Impairment of longitudinal fiber motion is therefore a sensitive and early marker of myocardial dysfunction, with prognostic value.^18^ This new modality has been applied during the last years in some studies for quantitative assessment of regional myocardial function of TM Patients, leading to interesting expected results.^1^,^6^,^19^ In fact these papers considered mostly patients with cardiac iron overload that were evaluated by echocardiograms performed at rest.

Conversely, in our work only 17.4% of patients had some evidence of cardiac iron and 38 of 46 (82.6%) had no evidence of cardiac iron overload. They underwent physical exercise to highlight by stress a contractile dysfunction that is not evident at rest, because this may potentially be an early sign of cardiomyopathy. The exercise capacity of this group of patients was comparable to that of healthy controls. The absence of ventricular hypertrophy and dilation may be due to the good clinical care, while dilation of the atria (50% of patients) is probably a consequence of residual anemia with transfusional regimens that keep the haemoglobin between 9.5 and 13.5 g%.

### Systolic function

As expected in patients whose iron overload cardiomyopathy is presumably in very early stages, the global and segmental LV systolic functions were normal at rest.

The most interesting data on the systolic function came from TDI. The average values of S’ waves measured on the basal septum and on the lateral mitral ring were normal at rest in TM. As expected, the S’ waves increased with exercise, in both groups, but in TM patients it achieved significantly lower values than in the control group (Figure 1).

**Figure 2:**
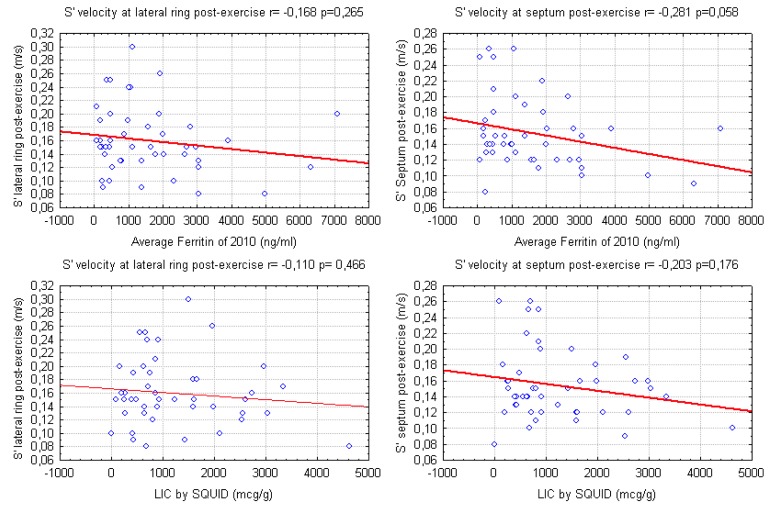
relations of S’ waves of the Left Ventricle (measured with Tissue Doppler Imaging after exercise) with mean serum Ferritin in the previous year (2010) and with Liver Iron Concentration measured by SQUID

**Figure 3:**
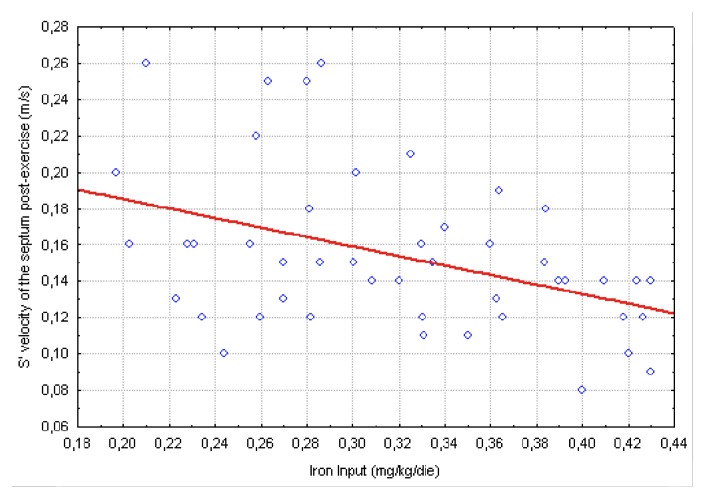
relation between iron input during the previous year (2010) and S’ waves measured by TDI on the basal segment of the septum after exercise (r= −0,413 p=0,004).

**Figure 4:**
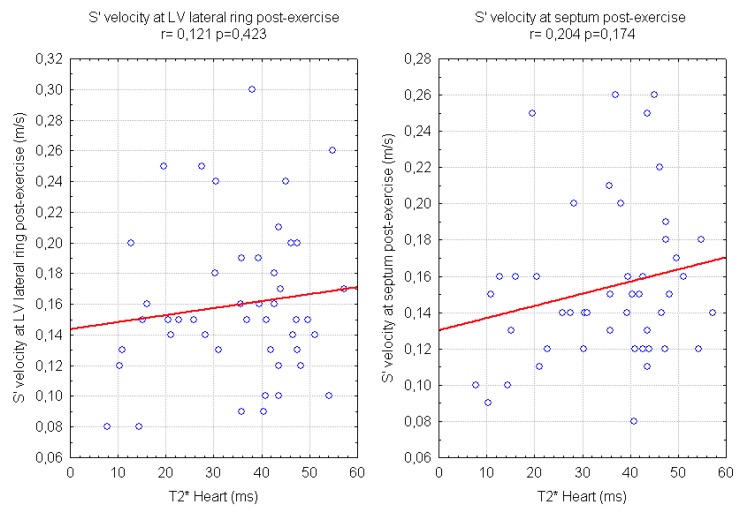
relations of echocardiographic tissue Doppler S’ waves of the Left Ventricle and Magnetic Resonance T2* time (value <20ms indicate iron overload).

Our data show a weak correlation between LV S’ waves values after exercise and LIC and also with mean ferritin values (Figure 2). A significant, negative, correlation of post-exercise S’ of interventricular septum with transfusional Iron Input was also shown (Figure 3). Finally we found a weak but interesting positive relation between LV S’ waves and heart T2* that decrease together (Figure 4).

Subgrouping patients according to the presence of cardiac iron at MRI, we did not found any significant difference in the echocardiographic values, except for TAPSE. Even for the TDI parameters of systolic velocity we did not showed a significant difference, although a trend to lower values was present in patients with T2 * <20 (Table 6).

The possible explanation for this finding could be that the group of patients with cardiac iron accumulation is too small to have sufficient statistical power and moreover the range of cardiac iron load is moderate-mild (7–19 ms with a mean of 13,4±3,7 ms). We should also keep in mind that some patients presenting without accumulation on MRI have had heart iron accumulation in the past then regressed after regular chelation therapy over the years. Finally, it is possible that the reduced systolic regional performance detected by TDI and likely to be attributed to iron damage, could be due to other factors, such as chronic anemia, that all patients with TM have in common.

Nevertheless, systolic S’ velocity at TDI evaluation done after a physical exercise were lower also in TM patients without evidence of cardiac iron overload when compared to a healty group: this is a new result, not yet described in TM patients, and further studies are needed to understand if the reduced shortening of longitudinal fibers could represent a very early sign of initial myocardial dysfunction.

The preservation of global systolic function could be explained by the patchy iron deposition in the heart,^20^ primarily involving the longitudinal subendocardial fibers. This may justify our observation of abnormal regional contraction that becomes evident in TM patients only during physical exercise, without any signs of global dysfunction. A recent study^21^ found that in TM patients, LV lateral systolic velocity was more reduced at rest in comparison to a control group, but no relation with iron overload parameters was investigated. Nevertheless, our observation that TDI waves are not reduced at rest confirms previous observational studies which found an inverse relation between TDI waves and iron overload.^1^ This emphasizes the utility of a diagnostic technique to demonstrate a possible iron-related dysfunction in patient considered at low risk because of a normal T2* value.

Conversely, in RV, systolic function - measured with TAPSE, FAC and S’ wave - is preserved, with a pattern similar to control group. The only difference we found is about the S’ wave measured at the tricuspid ring: under exercise, it increases more in TM patients than in control group. This difference recalls what described by Hamdy et al.^5^ using rest echocardiography. We hypothesize that this different response could be related to differences in the myosin isoforms between myocytes of the two ventricles,^22^ probably in response to increased pulmonary pressure that characterizes TM patients^23^ as in our study group. These facts could explain also the higher values of TAPSE we measured in TM patients with MRI evidence of iron load.

### Diastolic Function

Doppler analysis of flows through the AV valves shows normal average values of both mitral and tricuspid E/A ratio. The response to stress is normal, with filling velocities increase and non-significant decrease in E/A ratio bilaterally. According to Kremastinos et al.,^24^ also in our group of young asymptomatic patients, left ventricular compliance is normal, as is expected in well-treated patients in early stage of iron overload cardiomyopathy. On the other hand the lower tricuspidal E/A ratio, the longer isovolumetric relaxation time of the right ventricle and the higher PAPs in TM than in healthy controls suggest that the pulmonary circulation and the right heart are also affected by the disease since early stages, without any clinical symptoms. This aspect underlines the need of more focus on pulmonary hypertension and right heart function in thalassemia major. 16,23

## Limitations of this Study

We have not yet provided an adequate follow-up to validate the clinical impact of our findings. Because of the need to acquire data quickly after stopping the exercise, we could not conduct a full assessment of all myocardial segments to the TDI, but only the basal ones. However, these are generally considered the most reliable.^8^

## Conclusions

Our study shows that patients with beta thalassemia major, without clinical signs of heart failure and with normal EF under exercise stress may have a reduced response to exercise of left ventricle longitudinal fibers that could be detected with TDI echocardiography, even without MRI evidence of iron overload. To our knowledge this is a novel finding and we hypothesize that it may be a very early sign of initial myocardial damage by iron. Further investigations are warranted to confirm this hypothesis, in order to clarify if it could influence the clinical and therapeutic choices. A follow-up with the same technique could also confirm the stabilization, the exacerbation or regression of the dysfunction, and therefore the response to therapies.

From our data we conclude that stress echocardiography with TDI may be a non-invasive and repeatable imaging technique to assess the efficacy of chelation therapy and to identify early signs of myocardial involvement, even when MRI is not indicated or is negative for cardiac iron overload.

## Figures and Tables

**Table 1:** Characteristics of study population

	**Thalassemic** (n=46)	**Controls** (n=39)	***p*-value**
Age (years)	31,3±6	34,1±9,1	0,09
Sex (M/F)	29/17	22/17	
Weight (kg)	58,7±10,6	64,4±12,1	0,02
Stature (m)	1,62±0,07	1,70±0,07	0,002
BSA (m^2^)	1,62±0,16	1,74±0,19	0,003
HR (bpm)	84,6±13	68,5±6,7	<0,0001
SBP (mmHg)	114±17	117±12	0,29
DBP (mmHg)	69,24±9,35	73,25±10,59	0,23

BSA= body surface area, HR= heart rate, SBP= systolic blood pressure, DBP= diastolic blood pressure

**Table 2:** Hematological data of Thalassemic patients (n=46)

Ferritin (ng/ml)†	1596±1593
Hemoglobin (g/dl)†	11±0,5
Estimated value of Hemoglobin in the Echo’s day (g/dl)	11,5±1,1
Splenectomy (YES/NO)	30/16
Iron Input (mg/kg/die)†	0,32±0,07
Chelators:	
DFO	8
DFO+DFP	6
DFP	13
DFX	19
Compliance (%)	97,2±5,3
MRI-T2* of the Heart†	35,3±10,4
LIC by SQUID (mcg/g)†	1229±1027

† average of the year 2010;

**Table 3:** Comparison of echocardiographic data between thalassemic patients (n=46) and control group (n=39)

	Thalassemic	Controls	*p*-value
	
LV Mass (g)	77,6±16,3	88±24,3	0,02
LA Area (cm^2^)	19,8±3	16,8±1,4	<0,0001
LVEDD (mm)	47,9±4	49,2±6	0,25
LVESD (mm)	31,2±34,1	29,4±4,2	0,05
Indexed LVEDD (mm/m^2^)	29,7±3	28,2±1,6	0,008
IVS (mm)	8,1±1	9,2±1,4	0,0001
LVPW (mm)	7,8±0,8	8,6±1,2	0,0004
RA Area (cm^2^)	16,4±3	15,2±1,2	0,02
RVTS Area (cm^2^)	10,7±2,9	9,4±2,8	0,06
RVTD Area (cm^2^)	21,3±5,2	20,1±5,3	0,27

LA= left atrium; LVEDD= left ventricle end-diastolic diameter; LVESD= Left ventricle end-systolic diameter; IVS= interventricular septum; LVPW= left ventricle posterior wall; RA= right atrium; RVTS= right ventricle tele-systolic; RVTD= right ventricle tele-diastolic.

**Table 4:** Comparison of conventional echo-Doppler and TDI values of diastolic and systolic function at rest between thalassemic patients (n=46) and control group (n=39)

	Thalassemic	Controls	*p*-value
	
**DIASTOLE:**			
E mitral (m/s)	0,76±0,15	0,77±0,09	0,79
A mitral (m/s)	0,51±0,12	0,55±0,15	0,26
E/A mitral	1,55±0,41	1,47±0,3	0,35
EDT (msec)	176,9±28,9	183,97±37	0,47
E’ LV (cm/s)	18±3,6	17±3,2	0,26
A’ LV (cm/s)	9±2,8	9,8±3,8	0,19
E’ septal (cm/s)	13±3,2	11,5±3,3	0,008
A’ septal (cm/s)	9±1,9	9,7±3,2	0,04
E/E’ LV	4,3±0,98	4,6±0,89	0,17
E’/A’ LV	2,28±0,96	2,03±0,87	0,21
E tricuspid (m/s)	0,48±0,11	0,59±0,07	<0,0001
A tricuspid (m/s)	0,37±0,11	0,39±0,07	0,45
E/A tricuspid	1,4±0,4	1,56±0,21	0,025
E’ RV (cm/s)	15±3,3	15±3,2	0,78
A’ RV (cm/s)	13±4,2	10±3,2	0,0005
E/E’ RV	3,41±0,91	4,24±0,95	<0,0001
E’/A’ RV	1,29±0,5	1,65±0,58	0,004
IVRT (msec)	59,6±19,5	19,7±3,55	<0,0001
**SYSTOLE:**			
	
LVEF (%)	59,98±2,7	61,1±3	0,07
S’ septal (cm/s)	9±1,6	10±2,6	0,008
S’ LV (cm/s)	11±2,7	10±1,9	0,03
FAC RV (%)	50,2±5,9	52,8±6,1	0,04
S’ RV	14±2,4	15±3,8	0,11
TAPSE (mm)	23,9±3	24,2±3,6	0,74

LV= left ventricle; RV= right ventricle; FAC= fractional area change; EF= ejection fraction; EDT= deceleration time of mitral E wave; TAPSE= tricuspidal anulus plain systolic excursion.

**Table 5:** Comparison of conventional echo-Doppler and TDI values of diastolic and systolic function after maximal effort between thalassemic patients (n=46) and control group (n=39)

	Thalassemic	Controls	*p*-value
	
**DIASTOLE:**			
E mitral (m/s)	0,99±0,24	0,97±0,13	0,58
A mitral (m/s)	0,75±0,27	0,81±0,15	0,21
E/A mitral	1,43±0,43	1,23±0,27	0,02
E’ LV (cm/s)	22±5,1	24±4,7	0,16
A’ LV (cm/s)	13±4,8	19±3,9	<0,0001
E’ septal (cm/s)	18±4	22±5,2	0,0002
A’ septal (cm/s)	14±4,7	16±3,6	0,04
E/E’ LV	4,68±1,6	4,23±1,05	0,14
E’/A’ LV	2±0,8	1,36±0,5	<0,0001
E tricuspid (m/s)	0,64±0,22	0,71±0,13	0,1
A tricuspid (m/s)	0,52±0,17	0,63±0,19	0,006
E/A tricuspid	1,3±0,46	1,18±0,28	0,16
E’ RV (cm/s)	24±8,6	19±5,1	0,005
A’ RV (cm/s)	25±11	15±4,9	<0,0001
E/E’ RV	2,94±1,12	4±1,2	<0,0001
E’/A’ RV	1,07±0,5	1,45±0,66	0,004
IVRT (msec)	29,15±13,7	15,5±3,5	<0,0001
**SYSTOLE:**			
LVEF (%)	72,7±3,8	74,4±2,7	0,02
S’ septal (cm/s)	15±4,4	19±2,9	<0,0001
S’ LV (cm/s)	16±5	22±4	<0,0001
FAC RV (%)	56,2±6,4	59,4±6,44	0,02
S’ RV	22±4,2	16±3,8	<0,0001
TAPSE (mm)	30,5±4,3	29,2±3,5	0,08

LV= left ventricle; RV= right ventricle; FAC= fractional area change; EF= ejection fraction; EDT= deceleration time of mitral E wave; TAPSE= tricuspidal anulus plain systolic excursion.

**Table 6:** Comparison of TDI systolic velocity in patients with or without evidence of cardiac iron overload (defined as T2* < or > 20 ms)

	T2* < 20 ms (n=8)	T2* >20 ms (n=38)	*p-value*
**at rest:**			
S’ LV (cm/s)	10±2	11±3	0,42
S’ septal (cm/s)	8,5±1	9±1	0,35
**after exercise:**			
S’ LV (cm/s)	14,6±5,8	16,2±4,8	0,41
S’ septal (cm/s)	14,2±5,2	15,6±4,2	0,42

LV = Left Ventricle
